# The association between senior student tobacco use rate at school and alternative tobacco product use among junior students in Canadian secondary schools

**DOI:** 10.1186/1617-9625-12-8

**Published:** 2014-05-01

**Authors:** Adam G Cole, Scott T Leatherdale

**Affiliations:** 1 School of Public Health and Health Systems, University of Waterloo, 200 University Avenue, Waterloo, ON N2L 3G1, Canada

**Keywords:** Tobacco products, Adolescent, Tobacco use

## Abstract

**Background:**

The use of alternative tobacco products (ATPs) has grown in popularity among Canadian youth. This study examined the association between a school-level characteristic (the senior student tobacco use rate) and the current use of manufactured cigarettes, little cigars or cigarillos, cigars, roll-your-own cigarettes, smokeless tobacco (SLT), and a hookah among junior students.

**Methods:**

This study used nationally representative Canadian data from 29,495 students in grades 9 to 12 as part of the 2010/2011 Youth Smoking Survey. For each ATP, we described rates of senior and junior tobacco use, calculated the variance attributed to school-level factors, and examined the association between the senior student (grades 11 and 12) tobacco use rate and the current use of each ATP among junior students (grades 9 and 10) while accounting for relevant student-level characteristics. SAS 9.3 was used for all analyses.

**Results:**

Over half of schools sampled had senior students that reported using each ATP. School-level differences accounted for between 14.1% and 29.7% of the variability in ATP current use among junior students. Each one percent increase in the number of senior students at a school that currently use manufactured cigarettes, SLT, or a hookah was significantly independently associated with an increased likelihood that a junior student at that school currently used manufactured cigarettes (OR 1.04, 95% CI 1.01 to 1.06), SLT (OR 1.14, 95% CI 1.06 to 1.24), or a hookah (OR 1.09, 95% CI 1.03 to 1.14).

**Conclusions:**

Characteristics of the school environment a junior student attends appear to play an important role in ATP use, and tobacco control programs and policies should be designed to ensure that they include strategies to curb the use of all tobacco products. Additional evidence is needed for the impact of comprehensive school-based tobacco control approaches.

## Background

Over the past decade, the use of manufactured cigarettes among Canadian youth has decreased substantially [1]; however, evidence suggests that the use of alternative tobacco products (ATPs) such as roll-your-own tobacco, small cigars, and moist snuff has increased [2]. Because of the lower cost and appealing flavours of many of these products [3,4], youth may be enticed to try ATPs despite the health risks [5-8]. In addition, public health programs and policies that specifically address the prevention of use of ATPs are lacking (e.g., the Ontario Tobacco Control Strategy [9]). Given that there is little evidence identifying the factors associated with youth using ATPs, this is a domain that requires additional investigation.

Existing evidence indicates that youth who use ATPs and youth who use manufactured cigarettes share many characteristics, such as being male, older, and having more disposable income [10-12]. Furthermore, studies suggest that youth who use one tobacco product are more likely to use additional tobacco products [10-12]. Given that existing research indicates that junior students who attend schools with a higher prevalence of senior students that smoke manufactured cigarettes are more likely to smoke manufactured cigarettes [13], it is possible that a similar relationship may exist for ATPs. For instance, it would be informative to identify if junior students attending a school with a higher prevalence of senior students who use smokeless tobacco are more likely to use smokeless tobacco themselves. Confirming the presence of such an association would have important ramifications for targeting school-level prevention interventions given that youth spend a considerable amount of time at school where they can be influenced by tobacco control programming [13-16]. Moreover, considering that international data illustrate that comprehensive school-based tobacco control programs that prohibit the use of various tobacco products on school property have had a positive influence on the use of smokeless tobacco and manufactured cigarettes [17], this is a domain that warrants investigation in the Canadian context.

Therefore, the purpose of this study was to examine the association between a school-level characteristic (the senior student tobacco use rate) to the current use of five ATPs (little cigars or cigarillos, cigars, roll-your-own (RYO) cigarettes, smokeless tobacco (SLT), and hookah) among junior students in a nationally representative sample of secondary schools in Canada. Specifically, this study described the prevalence of senior students (grade 11 and 12) and junior students (grade 9 and 10) that use each ATP, calculated the variance in junior tobacco use rates that was attributable to school-level characteristics, and examined the association between the senior student tobacco use rate and the current use of ATPs among junior students while accounting for relevant student-level characteristics.

## Methods

### Sampling and recruitment

This cross-sectional study used representative data collected from 15,038 students in grades 9 and 10 and 14,457 students in grades 11 and 12 as part of the 2010/2011 cycle of the Canadian Youth Smoking Survey (YSS). The YSS is a self-reported questionnaire that students complete during class time; participants were not provided compensation. As described elsewhere [18], the target population for the data consisted of all young Canadian residents in grades 9 to 12 attending public, private, and Catholic secondary schools in 9 Canadian provinces. While New Brunswick participated in all prior cycles of the YSS, the provincial government chose not to participate in 2010/2011. Additionally, youth residing in Yukon, Nunavut, and the Northwest Territories were excluded from the target population, as were youth living in institutions or on First Nation Reserves and youth attending special schools or schools on military bases. The survey design and sample weight allow us to produce the population-based estimates within this manuscript. The University of Waterloo Office of Research Ethics and appropriate School Board and Public Health Ethics committees approved all procedures, including passive consent.

### Tobacco use

Current smoking status was measured by asking respondents if they have ever smoked 100 or more whole cigarettes in their lifetime, and on how many of the last 30 days they smoked one or more cigarettes. Consistent with Health Canada’s definitions of smoking status for the YSS [18] and the available measure of ATP use described below, *current manufactured cigarette smokers* had smoked at least one whole cigarette during the last 30 days preceding the survey. All other respondents were classified as *non-smokers*.

ATP use was measured using one multi-item question on alternate tobacco use. This question measured *current use* of each ATP among respondents: “In the last 30 days, did you use any of the following? (Mark all that apply)”, followed by a list of forms of tobacco other than cigarettes: cigarillos or little cigars (plain or flavoured), cigars (not including cigarillos or little cigars, plain or flavoured), roll-your-own cigarettes (tobacco only), smokeless tobacco (chewing tobacco, pinch, snuff, or snus), and water-pipe to smoke tobacco (also known as a hookah, sheesha, narg-eelay, hubble-bubble, or gouza). For this analysis, any respondents with all items missing had ATP current use set to missing.

### Student-level characteristics

The YSS also collected information on demographics, weekly spending money, and alcohol and marijuana use, which are important predictors of tobacco use. Similar to previous definitions [19,20], *non-drinkers* did not report alcohol use in the last year, *occasional drinkers* reported monthly alcohol use, and *current drinkers* reported weekly alcohol use. Similarly, *non-marijuana users* did not report marijuana use in the last year, *occasional marijuana users* reported monthly marijuana use, and *current marijuana users* reported weekly marijuana use.

### Senior student tobacco use rate

The senior student manufactured cigarette smoking rate for each school was calculated based on the number of current manufactured cigarette smokers in grades 11 and 12 (*senior students*) in the school, divided by the total number of senior students in the school. Similarly, the senior student little cigar or cigarillo, cigar, RYO cigarette, SLT, and hookah use rates for each school were calculated in a similar manner. In Quebec, the maximum grade in secondary school is grade 11; therefore, only grade 11 students were considered senior students in Quebec. All regression analyses only included grades 9 and 10 students (*junior students*) in the predictive models.

Schools were also classified as rural, suburban, or urban according to the population and population density obtained from the Statistics Canada website using the school’s postal code.

### Data analysis

Survey weights were used in the descriptive statistics of student-level characteristics to adjust for differential response rates across regions or groups. As described previously [18], the development of the survey weight was accomplished in two stages. In the first stage a weight (W_1j_) was created to account for the school selection within health region and school strata. A second weight (W_2jg_) was calculated to adjust for student non-response. The weights were then calibrated to the provincial gender and grade distribution so that the total of the survey weights by gender, grade and province would equal the actual enrolments in those groups.

Weighted descriptive analyses of the sample characteristics among junior students were examined according to tobacco product. The overall mean and range of senior student tobacco use rates were calculated, and unweighted analysis of variance statistics tested for significant differences in senior student tobacco use rates according to region and geographic classification. We then conducted two multilevel regression models per tobacco product among junior students using PROC GLIMMIX in SAS. The first model examined whether ATP use varied across schools through calculation of the intraclass correlation coefficient. The second model examined whether the senior student tobacco use rate was associated with the current use of each ATP while controlling for region, geographic classification, and various student-level characteristics. All analyses were performed using SAS 9.3 [21].

## Results

### Sample characteristics

Table 1 provides a summary of the number of included students by gender, grade, and region. Overall, there were no significant gender or grade differences in the number of junior students (χ^2^ = 1.19, df = 1, p > 0.05). Similarly, there were no significant gender or grade differences in the number of senior students (χ^2^ = 1.59, df = 1, p > 0.05).

**Table 1:** Summary of unweighted sample characteristics, 2010–2011, Canada

**Region**	**Gender**	**Grade, n (%)**			
		**9**	**10**	**11**	**12**
Atlantic^a^	Male	338 (4.4)	1181 (15.3)	1162 (15.1)	1060 (13.8)
	Female	381 (5.0)	1263 (16.4)	1243 (16.2)	1070 (13.9)
Quebec^b^	Male	277 (18.4)	223 (14.9)	240 (16.0)	n/a
	Female	292 (19.4)	233 (15.5)	237 (15.8)	n/a
Ontario	Male	894 (14.1)	767 (12.1)	730 (11.5)	665 (10.5)
	Female	956 (15.0)	881 (13.9)	793 (12.5)	670 (10.5)
Prairies^c^	Male	1034 (11.6)	1367 (15.4)	1174 (13.2)	943 (10.6)
	Female	910 (10.3)	1365 (15.4)	1160 (13.1)	929 (10.5)
British Columbia	Male	680 (13.5)	753 (14.9)	667 (13.2)	586 (11.6)
	Female	622 (12.3)	621 (12.3)	617 (12.2)	511 (10.1)
**TOTAL**	Male	3223 (10.9)	4291 (14.6)	3973 (13.5)	3254 (11.0)
	Female	3161 (10.7)	4363 (14.8)	4050 (13.7)	3180 (10.8)

Note: Data derived from the 2010–2011 National Youth Smoking Survey.

^a^Atlantic region includes Newfoundland and Labrador, Prince Edward Island and Nova Scotia.

^b^In Quebec, the maximum grade in secondary school is grade 11.

^c^Prairie region includes Manitoba, Saskatchewan and Alberta.

### Descriptive statistics for senior student tobacco use rates

**Figure 1:**
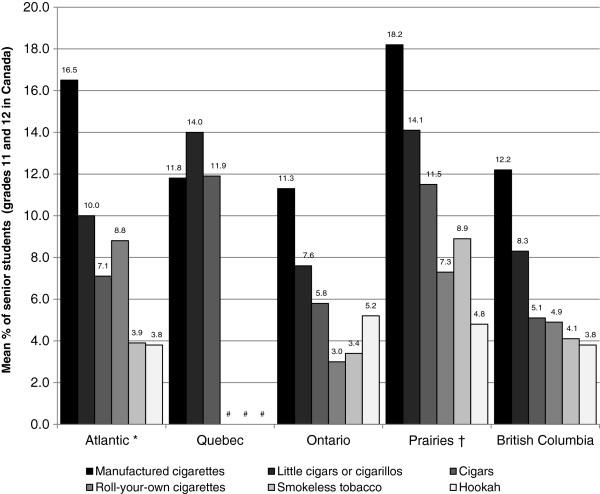
**Mean senior student (grade 11 and 12) tobacco use rate in secondary schools, by region, 2010-11, Canada.** Note: Data derived from the 2010-2011 National Youth Smoking Survey. * Atlantic region includes Newfoundland and Labrador, Prince Edward Island and Nova Scotia. † Prairie region includes Manitoba, Saskatchewan and Alberta. # Data suppressed due to high sampling variability.

As shown in Table 2, over half of schools sampled had senior students that reported currently using each product. The mean school senior student tobacco use rate varied across products: 5.5% (±4.0) of senior students within a school reported currently using a hookah, while 15.6% (±11.0) of senior students within a school reported currently using manufactured cigarettes. Figure 1 illustrates the mean senior student tobacco use rates across region. Overall, the mean senior student manufactured cigarette rate (F (4,133) = 6.40, p < 0.001), little cigar or cigarillo rate (F (4,133) = 2.93, p < 0.05), cigar rate (F (4,133) = 2.80, p < 0.05), RYO cigarette rate (F (4,133) = 8.43, p < 0.001), and SLT rate (F (4,133) = 10.32, p < 0.001) differed significantly across region.

**Table 2:** Summary of school-level senior student (grades 11 and 12) tobacco use rates, 2010–2011, Canada

**Tobacco product**	**Percent of schools with senior current users**^**a**^	**Senior student tobacco use rate (%)**^**b**^		
		**Mean (Std. Dev.)**	**Minimum**	**Maximum**
Manufactured cigarettes	91.3	15.6 (±11.0)	1.9	100.0
Little cigars or cigarillos	89.9	11.7 (±6.9)	1.7	33.3
Cigars	79.0	9.7 (±10.5)	1.0	100.0
Roll-your-own cigarettes	76.1	7.1 (±4.5)	0.5	20.4
Smokeless tobacco	60.1	5.9 (±5.0)	0.2	27.3
Hookah	66.7	5.5 (±4.0)	0.7	28.6

Note: Data derived from the 2010–2011 National Youth Smoking Survey.

^a^138 schools were identified with senior students (grades 11 or 12). Current manufactured cigarette users had smoked at least 100 cigarettes in their lifetime and smoked at least one whole cigarette during the past 30 days; all other current tobacco users had used the respective tobacco product at least once during the past 30 days.

^b^Excludes schools with no senior current users.

### Descriptive statistics for current tobacco product use among junior students

Overall, 10.9% of Canadian junior students reported currently using manufactured cigarettes. As shown in Table 3, there were no significant gender differences in the use of manufactured cigarettes among junior students (χ^2^ = 2.50, df = 1, p > 0.05), but more junior students in grade 10 reported currently using manufactured cigarettes compared to junior students in grade 9 (χ^2^ = 32.27, df = 1, p < 0.001). An estimated 5.9% of Canadian junior students reported currently using little cigars or cigarillos and 4.1% reported currently using cigars. As shown in Table 3, more male junior students reported currently using little cigars or cigarillos, and cigars compared to female junior students (χ^2^ = 83.4, df = 1, p < 0.001; and χ^2^ = 81.2, df = 1, p < 0.001, respectively), and more junior students in grade 10 reported currently using little cigars or cigarillos, and cigars compared to junior students in grade 9 (χ^2^ = 19.1, df = 1, p < 0.001; and χ^2^ = 6.6, df = 1, p < 0.05, respectively). Additionally, 3.4% of Canadian junior students reported currently using RYO cigarettes, 1.7% reported currently using SLT, and 2.5% reported currently using a hookah. As shown in Table 3, more male junior students reported currently using RYO cigarettes, SLT, and hookah compared to female junior students (χ^2^ = 34.8, df = 1, p < 0.001; χ^2^ = 83.7, df = 1, p < 0.001; and χ^2^ = 33.3, df = 1, p < 0.001, respectively), and more junior students in grade 10 reported currently using RYO cigarettes, SLT, and a hookah compared to junior students in grade 9 (χ^2^ = 13.2, df = 1, p < 0.001; χ^2^ = 11.8, df = 1, p < 0.001; and χ^2^ = 20.9, df = 1, p < 0.001, respectively).

**Table 3:** Weighted percent of current tobacco product use by demographic variables among junior students (grades 9 and 10), 2010–2011, Canada

**Parameters**		**Manufactured cigarettes**	**Little cigars or cigarillos**	**Cigars**	**Roll-your-own cigarettes**	**Smokeless tobacco**	**Hookah**
				**% of students**			
Gender	Female	10.5	4.0	2.6	2.4	0.7	1.7
	Male	11.3	7.6	5.5	4.2	2.6	3.2
Grade	9	9.4	4.9	3.6	2.8	1.3	1.8
	10	12.3	6.7	4.5	3.9	2.0	3.0

Note: Data derived from the 2010–2011 National Youth Smoking Survey.

**Figure 2:**
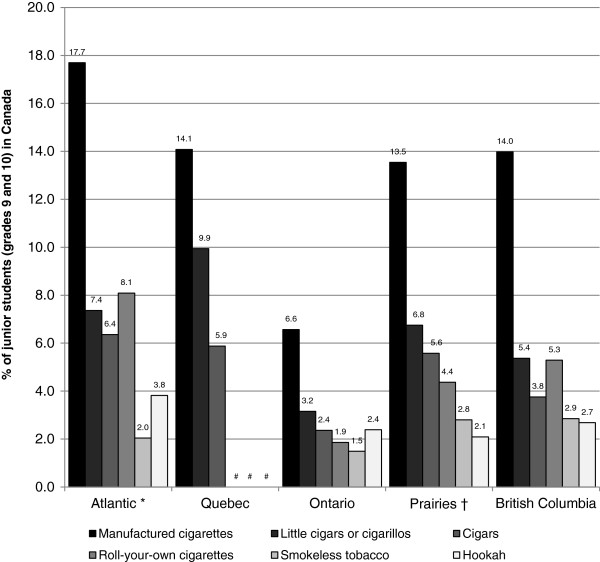
**Prevalence of current tobacco product use among Canadian students in grades 9 and 10, by region, 2010-11, Canada.** Note: Data derived from the 2010-2011 National Youth Smoking Survey. * Atlantic region includes Newfoundland and Labrador, Prince Edward Island and Nova Scotia. † Prairie region includes Manitoba, Saskatchewan and Alberta. # Data suppressed due to high sampling variability.

Figure 2 compares the prevalence of current use of each tobacco product among junior students in Canada by region. Compared to manufactured cigarettes, the use of other tobacco products is lower across all regions among junior students. The current use of little cigars or cigarillos is highest among junior students in Quebec (9.9%) and lowest in Ontario (3.2%), while the current use of SLT is highest among junior students in British Columbia (2.9%) and lowest in Ontario (1.5%). Additionally, the current use of cigars and RYO cigarettes is highest among junior students in the Atlantic region (6.4% and 8.1%, respectively) and lowest in Ontario (2.4% and 1.9%, respectively), while the current use of a hookah is highest among junior students in the Atlantic region (3.8%) and lowest in the Prairie region (2.1%).

### Between school variability in ATP use

Among Canadian junior students, significant between-school random variation in the odds of being a current little cigar or cigarillo, cigar, RYO cigarette, SLT, or hookah user were identified. As shown in Table 4, school-level differences accounted for between 14.1% and 29.7% of the variability in ATP current use among junior students.

**Table 4:** Intraclass correlation coefficients for current use of various alternative tobacco products among junior students (grades 9 and 10), 2010–2011, Canada

**Alternative tobacco product**	**σ**^**2**^_**μ0**_^**a**^	**Intraclass correlation coefficient**
Cigarillos or little cigars	0.613 (0.116)	0.157
Cigars	0.540 (0.118)	0.141
Roll-your-own cigarettes	0.945 (0.192)	0.223
Smokeless tobacco	1.391 (0.318)	0.297
Hookah	0.659 (0.157)	0.167

Note: Data derived from the 2010–2011 National Youth Smoking Survey.

^a^All models based on data from 138 secondary schools.

### Multilevel regression model results for the association between the senior student tobacco use rate and current ATP use among junior students

**Figure 3:**
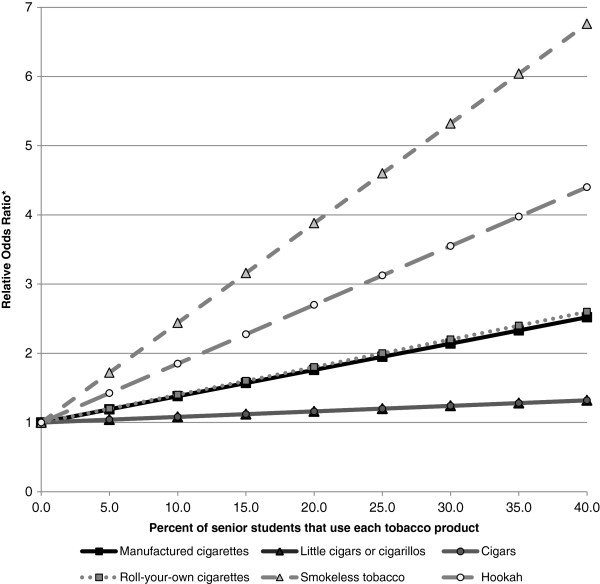
Summary of relative odds ratios of current tobacco use with each percent increase in the number of senior students that use each tobacco product, controlling for region, geographic classification, and relevant student-level characteristics*, 2010-11, Canada. Note: Data derived from the 2010-2011 National Youth Smoking Survey. * All models based on data from 133 secondary schools, controlling for region, geographic classification, and student-level characteristics (gender, grade, ethnicity, smoking status, parental smoking status, sibling smoking status, friend smoking status, disposable income, drinking status, and marijuana use status).

Table 5 presents a summary of the relative odds ratio estimates and corresponding p-values of current ATP use among junior students for each one percent increase in the number of senior students that currently use an ATP, controlling for region, geographic classification, and relevant student-level characteristics. It was identified that a one percent increase in the number of senior students at a school that currently use manufactured cigarettes was significantly associated with an increased likelihood that a junior student at that school currently used manufactured cigarettes (OR 1.04, 95% CI 1.01 to 1.06); a one percent increase in the number of senior students at a school that currently use SLT was also significantly associated with an increased likelihood that a junior student at that school currently used SLT (OR 1.14, 95% CI 1.06 to 1.24). Finally, a one percent increase in the number of senior students at a school that currently use a hookah was significantly associated with an increased likelihood that a junior student at that school currently used a hookah (OR 1.09, 95% CI 1.03 to 1.14). Although not statistically significant, each percent increase in the number of senior students at a school that currently use little cigars or cigarillos, cigars, or RYO cigarettes also modestly increased the likelihood that a junior student at that school currently used each of these products respectively. Figure 3 graphically illustrates the model-based estimated odds ratios of current tobacco product use among junior students for every percent increase in the prevalence of senior students at a school that currently use an ATP. For example, this figure illustrates that junior students that attend a school with an average senior student SLT use rate (5.9%) are almost twice as likely to currently use SLT compared to students that attend a school with no senior students that currently use SLT. In contrast, junior students that attend a school with the maximum senior student SLT use rate in this sample (27.3%) are almost five times as likely to currently use SLT compared to students that attend a school with no senior students that currently use SLT.

**Table 5:** Summary of relative odds ratios of current tobacco use among junior students (grades 9 and 10) for each percent increase in the number of senior students that use each tobacco product, 2010–2011, Canada

**Tobacco product**	**Relative odds ratio (95% CI)**^**a**^	**p-value**
Manufactured cigarettes	1.04 (1.01, 1.06)	0.002
Little cigars or cigarillos	1.01 (0.98, 1.04)	0.526
Cigars	1.01 (0.97, 1.05)	0.656
Roll-your-own cigarettes	1.03 (0.95, 1.11)	0.491
Smokeless tobacco	1.14 (1.06, 1.24)	<0.001
Hookah	1.09 (1.03, 1.14)	0.003

Note: Data derived from the 2010–2011 National Youth Smoking Survey.

^a^All models based on data from 133 secondary schools, controlling for region, geographic classification, and student-level characteristics (gender, grade, ethnicity, smoking status, parental smoking status, sibling smoking status, friend smoking status, disposable income, drinking status, and marijuana use status).

## Discussion

Alternative tobacco product (ATP) use is an important tobacco control issue among Canadian youth, and our results support the importance of the school environment to tobacco control initiatives. These data indicate that factors within the school environment accounted for up to 29% of the variability in ATP current use among junior students. The traditional research focus primarily on manufactured cigarettes has left a gap in our understanding where relatively little is known with respect to school-level factors that influence the use of ATPs, especially among youth. As a result, many current school-based tobacco control policies focus on the use of manufactured cigarettes and do not address the use of ATPs despite the known health risks. The lack of school-based policies that address the use of ATPs may be contributing to high rates of use, as our data illustrate that the majority of schools have senior students that use at least one tobacco product, and rates of tobacco use among senior students within a secondary school reach as high as 100%. It is clear that additional efforts are required to ensure that current and future tobacco control policies are not focused on a single product, but rather are designed to prevent the use of the broad range of tobacco products currently available in the marketplace. For example, the Smoke-Free Ontario Act, which prohibits smoking or holding lit tobacco at schools, could be amended to prohibit the use unlit tobacco products (such as SLT) at school [22].

These data provide additional support for the influence of older students to tobacco use among junior students. Figures 1 and 2 suggest that rates of tobacco use among senior students are mirrored among junior students: regions with high rates of tobacco use among senior students also have high rates of tobacco use among junior students. In fact, the results of the multilevel regression analyses show that high rates of SLT use and hookah use among senior students are independently associated with an increased likelihood that a junior student currently uses each of these products. This was especially true for SLT where a grade 9 or 10 student was approximately 5 times more likely to currently use SLT when at least 27% of senior students currently used SLT (the maximum senior student use rate in this sample). Since peers influence tobacco use [23,24] and students obtain tobacco products from each other [25,26], students may be exposed to non-traditional tobacco products through older students at the school. This has important implications for current and future school-based tobacco control policies. It is evident that inclusive tobacco control policies are necessary to ensure that students are not exposed to novel tobacco products through social influences in the school environment.

Numerous reasons have been identified to explain how a higher prevalence of senior student manufactured cigarette use influences junior student manufactured cigarette use, namely by increasing the acceptability of smoking behaviours [27,28], by increasing the availability of manufactured cigarettes at school [26,29], and by increasing the likelihood that a student has a friend that smokes manufactured cigarettes [30,31]. It is possible that senior students that use SLT or a hookah may influence junior students through a similar mechanism; however, future studies should explore the relationship between senior and junior tobacco users in order to inform future school-based tobacco control policies. Since there is evidence that school policies that prohibit the use of snus by students during school hours reduce the likelihood that a student uses snus [32], initiatives such as the Smoke-Free Ontario Act should be amended to prohibit the use of all tobacco products on and around school property, not just combustible products. In this way, the use of all tobacco products, including SLT, would be included in current tobacco control policies within the school context. The impact of this more comprehensive tobacco control approach would require evaluation.

Given the wide range of senior student tobacco use rates identified in this study (e.g., between 0% and 33% of senior students smoke little cigars or cigarillos in secondary schools), additional evidence is required to evaluate whether there are school policies in effect for the use of ATPs, whether these policies are consistently implemented and enforced, and what effect these policies have on the use of ATPs among students. Knowledge of these school policies will inform whether new school-based prevention and cessation programs are necessary or whether current school-based programs can be expanded to include the use of ATPs. Consequently, school-based policy evaluation tools (such as COMPASS; http://www.compass.uwaterloo.ca) that include questions that evaluate the existence and implementation of school-based programs and policies that aim to prevent the use of ATPs, represent an important domain of future research. Moreover, there may be a substantial benefit to future tobacco control prevention programming if researchers develop measures to identify the youth who are susceptible to using ATPs or multiple tobacco products, similar to the smoking susceptibility measure for manufactured cigarettes [33]. Measures of ATP susceptibility would allow researchers and practitioners to screen for youth who would be at the highest future risk for using ATPs and who should benefit the most from school-based ATP interventions.

The use of secondary data in this study presents a few limitations. Firstly, the current study relies on self-reported smoking behaviours; therefore the validity of responses cannot be guaranteed. However, self-report tobacco use measures have previously been demonstrated to be reliable and valid [34] and students were ensured that their responses were confidential. Secondly, measures of current use of tobacco products may not represent the usual use of these products by respondents and they do not provide any indication of the frequency of use. It is possible that a respondent first used a product once within the last 30 days; therefore this respondent would be classified as a current user, even though they are not a regular tobacco user. Thirdly, the cross-sectional nature of the data do not allow for the examination of how changes in the senior student smoking rates influence ATP use. Finally, it was outside of the scope of the current study to include school-level policy information which could impact senior student tobacco use rates. As a result, the relationship between school-level policies and the use of ATPs cannot be evaluated.

Despite these limitations, the present study has several strengths. First, the YSS is a nationally representative survey, providing insight to provincial differences in tobacco product use in Canada. Additionally, the YSS collects data on a range of tobacco products, producing the most comprehensive picture of tobacco use among youth in Canada. Moreover, this research expands on limited data for the influence of school-level characteristics to the use of ATPs. Finally, this research is the first to examine the influence of senior student tobacco use rates to the use of various ATPs.

## Conclusions

Tobacco use continues to be one of the most preventable causes of death and disability despite many public health programs and policies that discourage use. These data illustrate that characteristics of the school environment a student attends, such as the senior student tobacco use rate, appear to play an important role in ATP use among younger students in grades 9 and 10. Given the general positive association between the number of senior students that use ATPs and the likelihood that a junior student uses an ATP, additional evidence is required to examine the role of the school environment to the initiation and escalation of ATP use.

## Abbreviations

ATP: Alternative tobacco product; RYO cigarettes: Roll-your-own cigarettes; SLT: Smokeless tobacco.

## Competing interests

Both authors declare that they have no competing interest.

## Authors’ contributions

Both authors participated in the design of the study, analysis of the results, and helped to draft the manuscript. AC performed the statistical analysis. Both authors read and approved the final manuscript.
